# In Situ Polymerization of Polypyrrole @ Aluminum Fumarate Metal–Organic Framework Hybrid Nanocomposites for the Application of Wastewater Treatment

**DOI:** 10.3390/polym12081764

**Published:** 2020-08-07

**Authors:** Sarah Zayan, Ahmed Elshazly, Marwa Elkady

**Affiliations:** 1 Chemicals and Petrochemicals Engineering Department, Egypt-Japan University of Science and Technology, New Borg El-Arab City 21934, Alexandria, Egypt; ahmed.elshazly@ejust.edu.eg; 2 Chemical Engineering Department, Faculty of Engineering, Alexandria University, Alexandria 11432, Egypt; 3 Fabrication Technology Department, Advanced Technology and New Materials and Research Institute, City of Scientific Research and Technological Applications (SRTA-City), New Borg El-Arab City 21934, Alexandria, Egypt

**Keywords:** aluminum fumarate, MOF, polypyrrole, nanocomposite, functionalization

## Abstract

Composite metal–organic frameworks combine large and accessible surface areas with low density and high stability. Herein, we present novel nanocomposites of polypyrrole/aluminum fumarate metal–organic framework (PPy/AlFu MOF), which were synthesized via in situ oxidative polymerization with the aim of MOF functionalization to enhance its thermal stability and increase the specific surface area so that these nanocomposites may be used as potential adsorbents. The synthesized nanocomposites were characterized by various techniques, such as powder X-ray diffraction, scanning electron microscopy, and Fourier-transform infrared spectroscopy (FTIR). The successful functionalization of aluminum fumarate MOF was confirmed by FTIR, and the Brunauer–Emmett–Teller (BET) surface area of the PPy/MOF nanocomposite slightly increased from 795 to 809 $m^{2}/g$. Thermogravimetric analysis data also show that the weight loss of the composite is up to 30% at temperatures up to 500 ℃. Remarkably, lead (50 ppm) sequestration using the composite was tested, and the atomic absorption spectrometry data demonstrate that PPy/MOF is a super-adsorbent for heavy metal ions. This work shows that the novel polymer–MOF composites are promising materials for the selective removal of lead from wastewater streams.

## 1. Introduction 

Metal–organic frameworks (MOFs) are a class of crystalline, porous solids composed of organic ligands connected by inorganic secondary building units (SBUs). These solids are renowned for their uniform and highly tunable pore structures, along with enormous variability in inorganic units’ structure and linker topology, connectivity, and chemical functionality [1,2]. Conversely, it is also known that various types of MOFs have the potential to lose structural integrity in an aqueous medium, which hinders their use in potential applications such as adsorption [3,4] and water desalination [5,6]. However, the MOFs containing zirconium, iron, and aluminum metal ion clusters show reasonable stability in water applications [7]. In recent years, polymer–MOF composites have appeared in literature because they are thought to combine the control of conductive polymer chains’ functionality and the enhanced surface area of MOFs [8,9]. 

Metal oxides represent one type of adsorbent that has been investigated widely for heavy metal removal by adsorption, with nanosized metal oxides, which are characterized by their high specific surface areas and high activity, being of particular interest. However, metal oxides have issues associated with their stability due to their nanoscale size, which leads to particle aggregation and subsequent drop in removal efficiencies [10]. Zeolites represent another class of adsorbent that is efficient and widely applied in adsorption. There are over forty types of natural zeolites and more than a hundred synthetic varieties. Zeolites have moderate surface areas, uniform pores, and an overall negative charge. Natural zeolites can be modified to improve their effectiveness for heavy metal removal from water by applying chemical treatment [11]. 

The unique characteristics of metal–organic frameworks have distinguished them as unique adsorbent materials because they possess diverse active sites, flexibility of pore topology, well-defined crystallinity, large pore volume, good thermal stability, and excellent selectivity as adsorbents. Recently, there has been much research on the synthesis and application of MOFs. Some studies focused on the synthesis of mesoporous MOFs and potential adsorption applications [12,13], while others concerned the decontamination performance of different pollutants by MOFs, such as heavy metal pollutants [14], toxic and radioactive metal ions [15], dyes [16], and aromatic pollutants [17]. Other researchers focused on adsorption mechanisms [18,19].

Moreover, the outstanding properties of metal–organic frameworks (MOFs) with various structural patterns that are derived from a combination of inorganic metallic nodes and organic linkers have attracted intense research work in both academia and industry. In terms of MOFs’ applications, a recent essential challenge has been that of providing low-density porous materials with relatively high stability, low poisonous/harmful potential, and superior performance [20]. Lightweight alkali earth metals such as calcium [21], aluminum, and magnesium attract intense focus as inorganic nodes in metal–organic frameworks. Among them, aluminum is the most successful candidate because it allows the formation of several one- or two-dimensional inorganic networks, which lead to a wide variety of aluminum-based organic frameworks. Thus, aluminum–organic frameworks are promising materials for academic research and industrial applications [22].

Indeed, the BASF SE company has optimized a synthesis technique to produce organic-solvent-free aluminum–organic frameworks by using water as a clean solvent, which leads to a rise in the production yield while also being an economic and eco-friendly technique. In brief, the green method includes replacing aluminum nitrate or chloride salts with aluminum sulfate to avoid toxicity and corrosion, respectively. Water is used as a reaction medium instead of dimethylformamide, leading to a significant surge in the Basolite A520 yield. Finally, the addition of a strong base, such as sodium hydroxide, allows for dissolving the organic linker in an aqueous solution to produce a homogeneous reaction medium [20,22].

**Figure 1:**
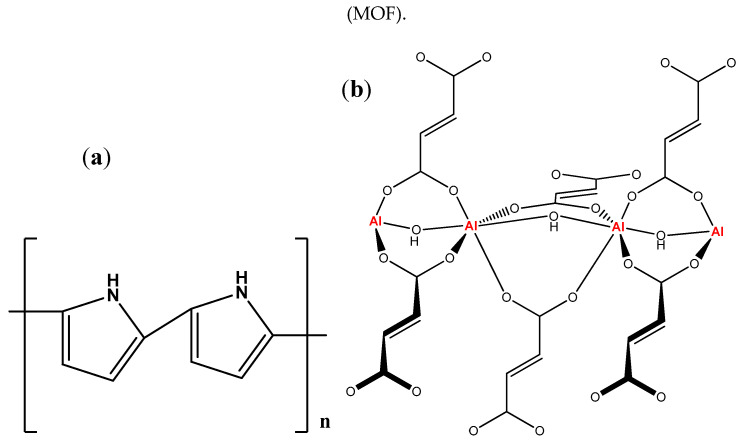
Chemical structure of (**a**) polypyrrole and (**b**) aluminum fumarate MOF.

Conductive polymers such as polypyrrole, shown in Figure 1a, and their composites have various applications in the sensors industry and wastewater treatment as adsorbents; this is because polypyrrole has remarkable electrical and thermal stability, exceptional mechanical properties, and notable adsorption capacity to remove heavy metals from water effluents [23]. Moreover, it has an effortless combination and adaptability in handling.

**Figure 2:**
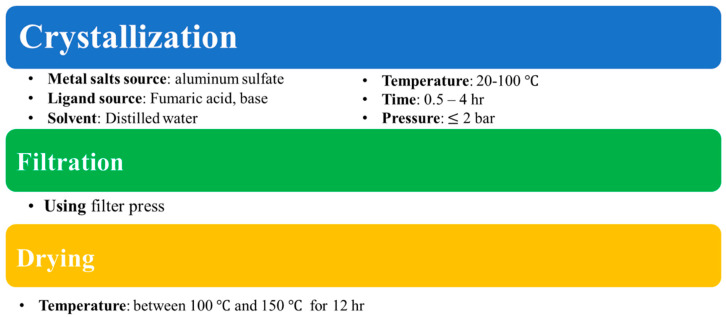
Block diagram of the green synthesis of aluminum fumarate metal–organic framework.

Herein, we account for bottom-up fabrication of polypyrrole/aluminum fumarate metal–organic framework (PPy/AlFu MOF) nanocomposites based on the excellent thermal stability and immense heavy metal removal efficiency of polypyrrole and high specific area of MOFs. Primarily, aluminum fumarate MOF, Figure 1b, was synthesized by the green reaction process using water as a solvent [24], illustrated in the block diagram in Figure 2. Then, polypyrrole underwent in situ chemical oxidative polymerization on the surface of the aluminum fumarate MOF, with ferric chloride as an oxidizing agent. The enhancement of thermal stability of the composite was confirmed by thermogravimetric analysis, while the rise in composite surface area was measured by Brunauer–Emmett–Teller (BET) analysis. The results of atomic absorption analysis demonstrate that the PPy/MOF composite could be a promising candidate for lead removal due to its good porosity and adsorption capacity. 

## 2. Materials and Methods 

### 2.1. Materials

Aluminum sulfate octadecahydrate (97%), fumaric acid (≥99%), sodium hydroxide (reagent grade, ≥98%), pyrrole (reagent grade, 98%), ferric chloride (reagent grade, 97%), nickel chloride (98%), and manganese chloride (99%) were purchased from Sigma Aldrich and used without further purification. 

### 2.2. In Situ Polymerization of Pyrrole with Aluminum Fumarate MOF

Initially, neat aluminum fumarate MOF was prepared by the green synthesis process [14]. Three grams of aluminum sulfate octadecahydrate was dissolved in 15 mL distilled water, and then the solution was added dropwise to a solution of 5 mL NaOH (50 wt.%) with 1 g fumaric acid dissolved in 15 mL distilled water. After that, the mixture was stirred for an hour at 90 ℃, while a snow-white precipitate was formed and separated at high-speed centrifuge. Neat polypyrrole was fabricated according to the procedure found in [23,25]. 

The obtained aluminum fumarate MOF was thermally treated at 100 ℃, then added to highly diluted hydrochloric acid (0.01 M, 30 mL) to chemically activate the MOF surfaces. After that, thermally and chemically treated MOFs were dried. The procedure for the synthesis of the PPy/MOF nanocomposite started with adding pyrrole (3 mmol) to a sonicated solution of HCl (0.01 M, 30 mL) containing AlFu MOF (0.1 g). At this point, the solution was sonicated for half an hour to disperse the pyrrole monomer in the reaction media and allow it to be wholly adsorbed to the surface of MOFs. After that, the sonicated solution was put in an ice bath and stirred at low speed. Then, the dopant solution (0.01 M HCl, 30 mL) containing ferric chloride (0.2 g) was added dropwise into the suspension solution. Stirring continued for 4 h, and then the final black product was filtered and washed with distilled water to remove unreacted monomers. After drying at 60 ℃ for 24 h, the final black powder PPy/MOF nanocomposite was obtained. 

### 2.3. Characterization 

The surface morphologies were investigated by scanning electron microscopy (SEM), while the specific surface areas were calculated from nitrogen adsorption/desorption isotherm data conducted at 77 K. Thermal stability was measured by a thermogravimetric analysis technique (TGA). Moreover, structural characterization was confirmed by X-ray diffraction (XRD), and the functional groups were determined by Fourier-transform infrared spectroscopy (FTIR). 

### 2.4. Determination of Optimum pH and Composite Dose

The batch adsorption experiments were carried out using a magnetic stirrer at 200 rpm. Initially, the optimum composite dosage was determined by using composite dosage from 0.5 to 3 g/L at pH 7 and an adsorption time of 24 h at room temperature for lead feed concentration of 50 ppm. After optimum composite dosage selection, another several adsorption experiments took place at various pH from 3 to 9 under the same conditions as previously mentioned. The pH of the lead solution during adsorption was measured using a multimeter.

### 2.5. Adsorption Study 

The removal efficiencies of the composite to ${\mathrm{Pb}}^{2+}$ ions in aqueous solution were studied using a batch adsorption technique. Solutions were loaded with 50 ppm ${\mathrm{Pb}}^{2+}$ ions by dissolving ${\mathrm{PbCl}}_{2}$ in distilled water. Then, 0.1 g PPy/MOF composite powder was immersed in 250 mL of solution for about three hours at room temperature. Solution samples of 5 mL were withdrawn at different time intervals, after which the atomic absorption analysis technique was used to measure the residual concentration of ${\mathrm{Pb}}^{2+}$ ions. ${\mathrm{Pb}}^{2+}$ adsorption onto the composite at each exposure time, $q_{t}$ (mg/g), was derived as follows [16]:

(1) $$q_{t}=\frac{\left(C_{i}-C_{t}\right)\;V}{m}$$

where $C_{i}$ is the initial ${\mathrm{Pb}}^{2+}$ concentration (mg/L), $C_{t}$ is the concentration at time t (mg/L), V is the volume of solution (L), and m is the mass of the composite sample (g).

### 2.6. Lead Adsorption Isotherm

Lead adsorption isotherm was determined by adding the optimum concentration of PPy/MOF with various lead feed solutions (250 mL each) in Erlenmeyer flasks, with concentration varying from 200 to 1000 ppm at optimum pH. The composite was kept in the lead solution and stirring took place at 200 rpm until equilibrium was achieved. 

The amount of lead adsorbed on the composite was calculated by following mass balance Equation (2), where Q_e_ is the amount of lead adsorbed per gram of composite; C_0_ and C_e_ are the initial and equilibrium concentration of lead ions in solution, respectively; V is the volume of solution; and W is the weight of PPy/MOF.

(2) $$Q_{e}=\frac{\left(C_{0}-C_{e}\right)\times V}{W}$$

The obtained results from the equation were fitted into two different isotherms, namely Langmuir and Freundlich, to understand the nature of adsorption. The Langmuir isotherm is shown in Equation (3).

(3) $$Q_{e}=\frac{{\mathrm{KQ}}_{m}C_{e}}{1+{\;\mathrm{KC}}_{e}}$$

where C_e_ is the equilibrium concentration of lead in the solution (ppm), Q_e_ is the amount of lead adsorbed per unit mass of composite (mg/g), Q_m_ is adsorption capacity (mg/g), and K is adsorption equilibrium constant (L/mg). Similarly, the Freundlich isotherm is shown in Equation (4).

(4) $$Q_{e}={\;K}_{f}C_{e}^{\frac{1}{n}}$$

where *n* and $K_{f}$ are Freundlich constants. 

The same study was carried out at two different temperatures, 298 and 308 K, to better understand the effect of temperature on adsorption.

### 2.7. Effect of Coexisting Heavy Metals

Different heavy metals ions such as iron (${\mathrm{Fe}}^{3+}$),  nickel (${\mathrm{Ni}}^{2+}$),  and manganese (${\mathrm{Mn}}^{2+}$) were taken into account to study the interference of these ions with adsorption of lead by the PPy/AlFu MOF composite. Different stock solutions were prepared at 298 K with a lead concentration of 50 ppm at pH 7. The concentration of co-ions varied from 100 to 500 ppm for iron, nickel, and manganese ions. The amount of adsorbent dosed was 1.5 g/L for each adsorption experiment. 

### 2.8. Lead Adsorption of PPy, MOF, and Composite

To compare the removal efficiencies of polypyrrole, aluminum fumarate MOF, and PPy/MOF composite, lead (50 ppm) adsorption experiments were conducted for two hours; every 30 min, a sample was collected and sent to atomic absorption spectroscopy (AAS) to analyze the lead concentration. All the experiments were done at room temperature and pH 7, with adsorbent dosage equal to 1.5 g/L. 

### 2.9. Regeneration Study

The spent PPy/AlFu MOF composite was subjected to desorption using sodium hydroxide solution as a regenerator. Initially, the composites used in all batch adsorption experiments were collected and filtered using a funnel before being dried at 100 ℃ for three hours. The dried composite was then weighed and added to different concentrations of sodium hydroxide solution (0.5–1.5 M) at a dosage of 1.5 g/L. The residual lead concentration was measured after fixed intervals of 0, 1, 3, 5, and 7 h using atomic absorption spectroscopy, and the percentage of desorbed lead was calculated according to the equation

(5) $$D\%\;=\frac{C_{\mathrm{des}}}{C_{\mathrm{ads}}}\times 100\%$$

where D is the desorbed percentage, $C_{\mathrm{des}}$ is the desorbed concentration, and $C_{\mathrm{ads}}$ is the adsorbed concentration at equilibrium.

## 3. Results and Discussion

### 3.1. Characterization of Polypyrrole/Aluminum Fumarate MOF Composite

**Figure 3:**
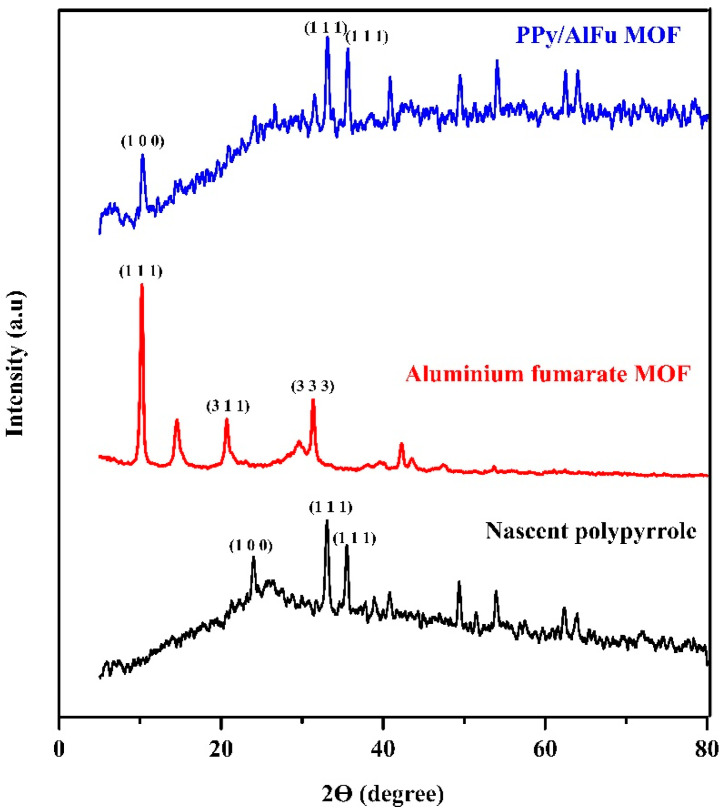
XRD pattern.

To better explore the formation of PPy/MOF composites, the X-ray diffraction (XRD) patterns were investigated. Figure 3 suggests that the neat aluminum fumarate MOF had the highest crystallinity and that the intensity of the characteristic peaks in the neat materials were stronger than those of the composite (2θ = 10 and 33°, which matched with their indices of (100) and (111), respectively) [23,26]. The crystal size of PPy/AlFu MOF was calculated using Scherrer’s equation, giving an average crystal diameter of 18.6 nm. Whereas the presence of PPy in the composite decreases the degree of crystallinity of MOF in the composite, the characteristic peaks of both polypyrrole and aluminum fumarate MOF were still observed in the composite. It might be concluded that there were physical interactions (physisorption) between polypyrrole, which has positively charged sites [27], and the negatively charged MOF surfaces [26].

**Figure 4:**
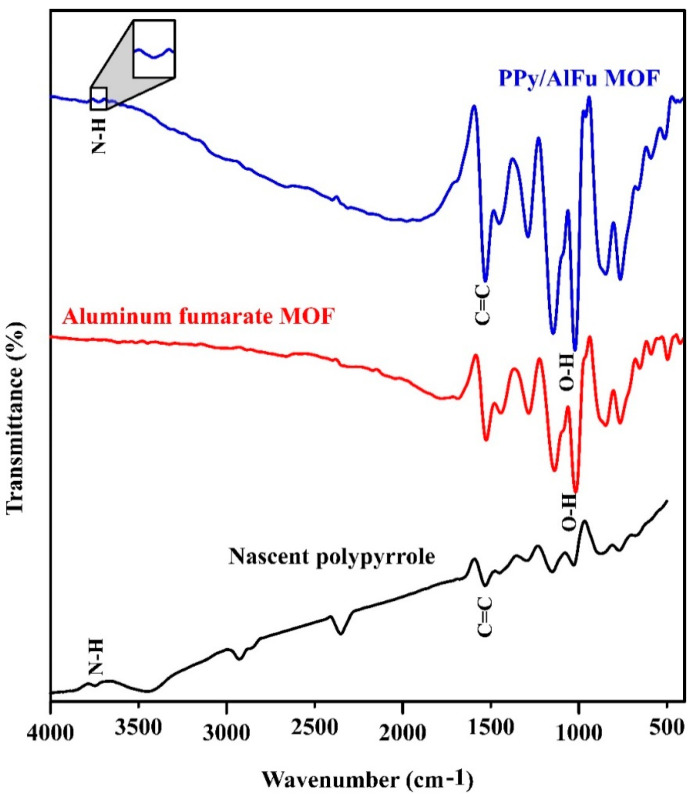
FTIR spectroscopy.

The chemical structures of the neat polypyrrole, aluminum fumarate MOF, and PPy/MOF composites were characterized by FTIR, and the results are shown in Figure 4. Regarding neat polypyrrole, the bands obtained at 1530 and 3700 ${\mathrm{cm}}^{-1}$ are attributed to the characteristic peak C=C stretching and N-H stretching vibrations, respectively [28]. The peaks observed at 950 ${\mathrm{cm}}^{-1}$ correspond to O-H bending in aluminum fumarate MOF [29]. Considering the PPy/MOF composite, the successful in situ polymerization of polypyrrole at the surface of MOF was also confirmed through O-H stretching at 930 ${\mathrm{cm}}^{-1}$, besides the presence of N-H peak at 3750 ${\mathrm{cm}}^{-1}$, which is characteristic of polypyrrole. 

**Figure 5:**
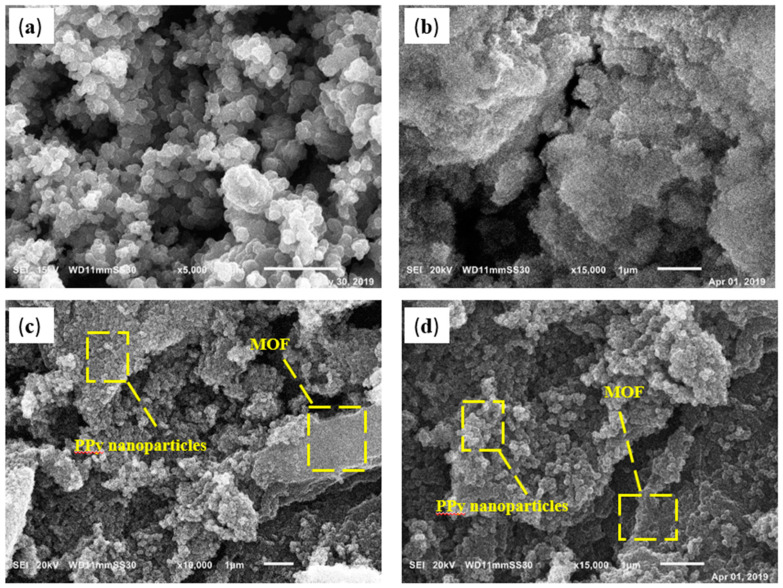
SEM images: (**a**) polypyrrole (PPy) particles, (**b**) MOF, and (**c**,**d**) PPy/MOF composites.

Figure 5 demonstrates the surface SEM morphologies of pure polypyrrole, neat aluminum fumarate MOF, and PPy/MOF composites with two different magnifications. Figure 5a,b represent polypyrrole with average particle size equal to 190 nm and aluminum fumarate MOF, respectively. Figure 5c,d display the PPy/MOF composite where numerous polypyrrole nanoparticles, with average particle size equal to 135 nm, are randomly distributed on the MOF surface. After the in situ polymerization, the composite maintains its original structure, which confirms the surface adhesion between PPy and MOF. In addition, the polymerized polypyrrole could be coated on the surface of MOFs without blocking the MOF pores [30]. 

**Figure 6:**
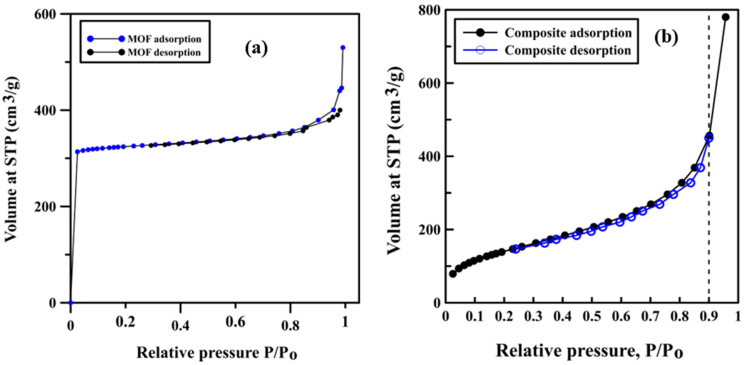
(**a**) MOF isotherm; (**b**) PPy/MOF composite isotherm.

The surface areas of AlFu MOF and the composites were obtained from nitrogen gas adsorption isotherm by Brunauer–Emmett–Teller (BET) analysis at 77 K. MOF and the composites were grounded to avoid agglomeration and then degassed at 200 and 350 ℃, respectively, under vacuum to remove any gases from the pores. Figure 6a shows that the MOF isotherm is a Type I isotherm with a steep rise at P/P0 < 0.1 followed by approximately constant adsorption at higher pressure; this isotherm significantly matches with the one reported in the literature [26]. Figure 6b shows that the adsorption/desorption isotherm of the PPy/MOF composite is also a Type I isotherm, and the presence of polypyrrole at the surface of MOF does not affect the adsorption behavior. Moreover, the maximum adsorption capacities of neat MOF and PPy/MOF composite at P/P0 equal to 0.9 are slightly less than 400 ${\mathrm{cm}}^{3}/g$ and approximately 470 ${\mathrm{cm}}^{3}/g$, respectively. The rise in adsorption loading is due to the enhancement of the MOF’s capacity by embedding polypyrrole on its surface, which leads to an increase in the BET surface area of the composite. BET surface areas were equal to 795 $m^{2}/g$ for the nascent MOF and 809 $m^{2}/g$ for the composites. In addition, at low pressures, the composite uptake is lower than the MOF uptake. 

Interestingly, the polymerization of pyrrole takes place at the external surface of MOF, which leads to the integration of the areas without blocking the MOFs’ pores, which appears in the slight rise of BET area of MOF. 

**Figure 7:**
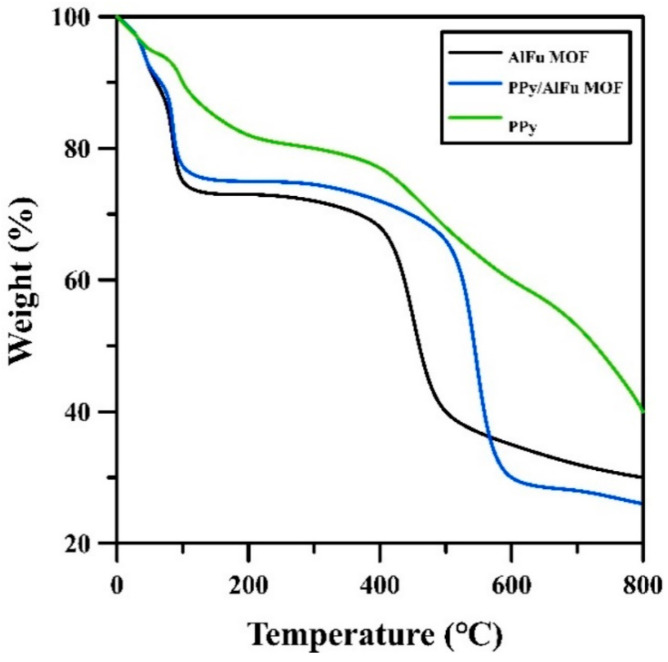
TGA curve.

The change in the weight of polypyrrole, aluminum fumarate MOF, and polypyrrole/MOF composites with the increase of temperature was studied by thermogravimetric analysis in temperature range between 25 and 800 ℃, and the results are shown in Figure 7. Both MOF and composite followed the same pattern of losing weight with increasing temperature, while polypyrrole followed another behavior. PPy exhibited weight loss of about 13% up to 200 ℃, which may have been due to the evaporation of adsorbed water and low-molecular-weight volatiles. A considerable weight loss, up to 30%, was then noticed at the temperature up to approximately 500 ℃ [31]. 

A relatively significant weight loss takes place in the neat MOF and the composites due to the vaporization of adsorbed volatile components inside the pores and on the surface of the particles. This vaporization is observed at temperatures between ambient temperature and approximately 100 ℃. After that, a weight loss of about 27% took place in the neat MOF in the temperature range between 100 and 370 ℃ [26]. Moreover, a composite weight loss equal to 23% was achieved in the temperature range between 100 and approximately 470 ℃. 

### 3.2. Adsorption Study 

**Figure 8:**
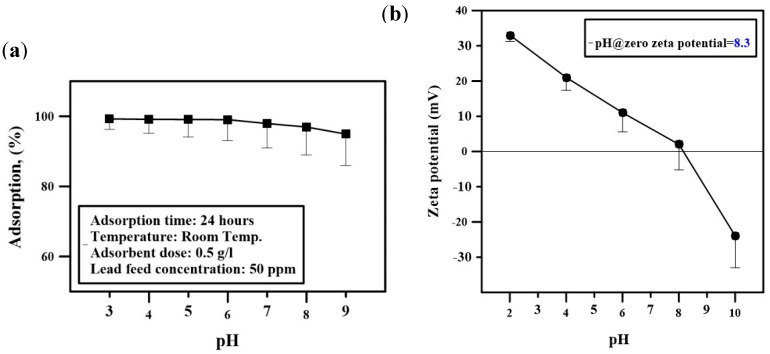
Effect of pH on (**a**) adsorption efficiency and (**b**) zeta potential.

**Figure 9:**
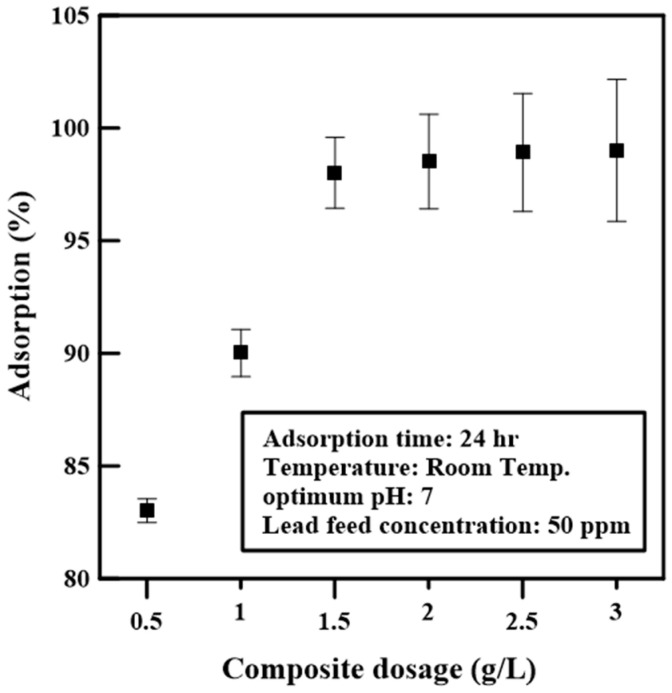
Effect of composite dosage on adsorption efficiency.

The results of the study of the effect of changing the pH and the composite dosage during adsorption experiments are presented in Figure 8a and Figure 9. It is noticed from Figure 8a that the adsorption efficiency is negatively affected at high pH and started decreasing after pH 8.3. This is because composite material after pH 8.3 becomes negatively charged, which is confirmed by studying the change of zeta potential with changing pH. Zero zeta potential was obtained at pH 8.3, and the lower the pH, the more the composite is negatively charged, which facilitates the adsorption of heavy metals due to physical adsorption between counter-charged particles. The range of pH studied was from 3 to 9. The adsorption removal efficiency is approximately 100% in the pH range 3–7, whereas the isoelectric point of the composite is at pH 8.3, as shown in Figure 8b. All the adsorption experiments were carried out at neutral pH. 

The optimum dosage of the PPy/MOF composite was obtained by carrying out adsorption experiments at neutral pH with a composite dosage range of 0.5–3 g/L. In Figure 9, the data show that 1.5 g/L is good enough to reach approximately 100% lead removal with the feed concentration of 50 ppm. Thus, the adsorbent dose of 1.5 g/L was chosen for use in the rest of the experiments. 

### 3.3. Adsorption Isotherms 

**Figure 10:**
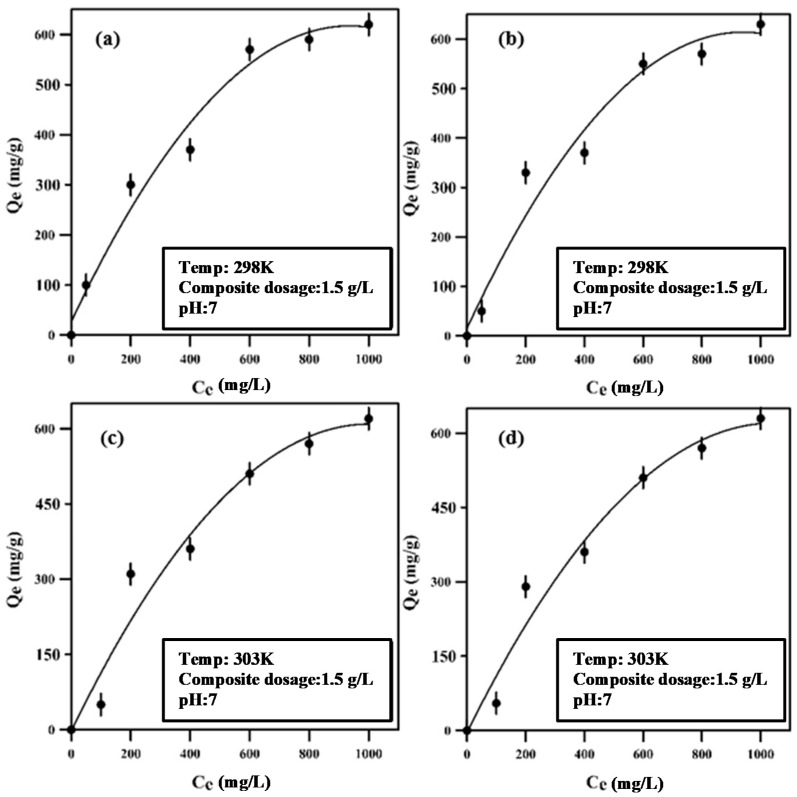
Langmuir isotherm (**a**,**c**); Freundlich isotherm (**b**,**d**).

Lead maximum feed concentration was taken to be 1000 ppm for the synthetic solution. Figure 10 displays the Langmuir and Freundlich isotherms at two different temperatures: 298 and 308 K. The data fitting of the two isotherms shows that the Langmuir isotherm gives better data fitting than the Freundlich isotherm. The figure also shows that the rise in temperature leads to a drop in the maximum adsorption capacity of the composite, which makes sense for an exothermal adsorption process. The data from both isotherms indicate that the maximum adsorption capacity of the composite is approximately 600 mg/g at neutral pH and room temperature (298 K). Table 1 presents the Freundlich and Langmuir constants at 298 and 308 K. 

The adsorption performance of the fabricated PPy/MOF composite was compared with the adsorption performances of various previously investigated low-cost activated-carbon-based materials in terms of adsorption capacity (Q_e_). Table 2 compares the adsorption capacity of synthesized PPy/MOF with various studied low-cost activated carbons. It can be seen that the adsorption capacity of the PPy/MOF composite is the highest when compared with other previously studied activated carbon materials. 

### 3.4. Effect of Coexisting Heavy Metals

**Figure 11:**
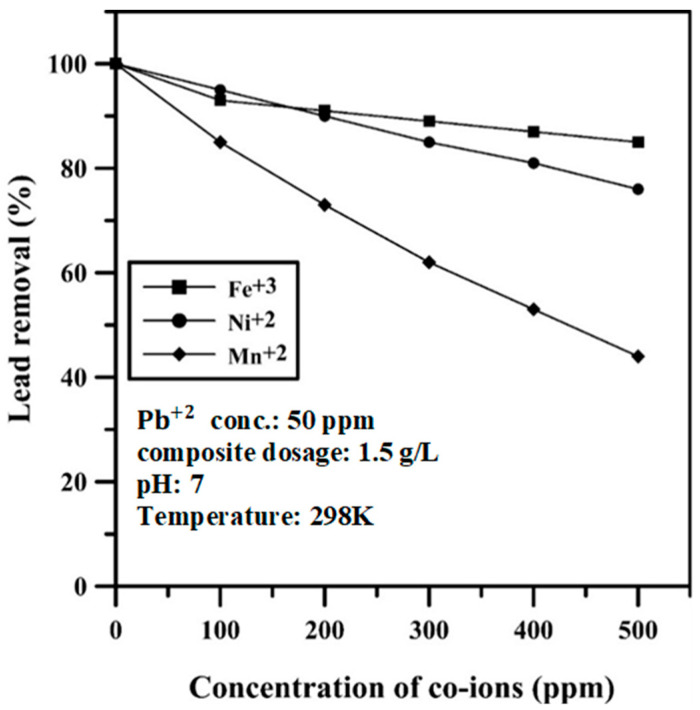
Effect of coexisting heavy metals ions.

The effect of the existence of three hazardous heavy metals, namely, iron, nickel, and manganese is presented in Figure 11, as well as the adsorption experiments’ conditions. The figure shows that the presence of iron at high concentration does not affect the adsorption efficiency of lead. Conversely, manganese ions in the adsorption medium lead to a significant reduction in the sequestration efficiency of lead. With the incorporation of manganese ions, the lead ion removal drops by about 50%, whereas the reduction in nickel removal is not a significant drop, about 15%. 

### 3.5. Lead Adsorption of PPy, MOF, and Composite 

**Figure 12:**
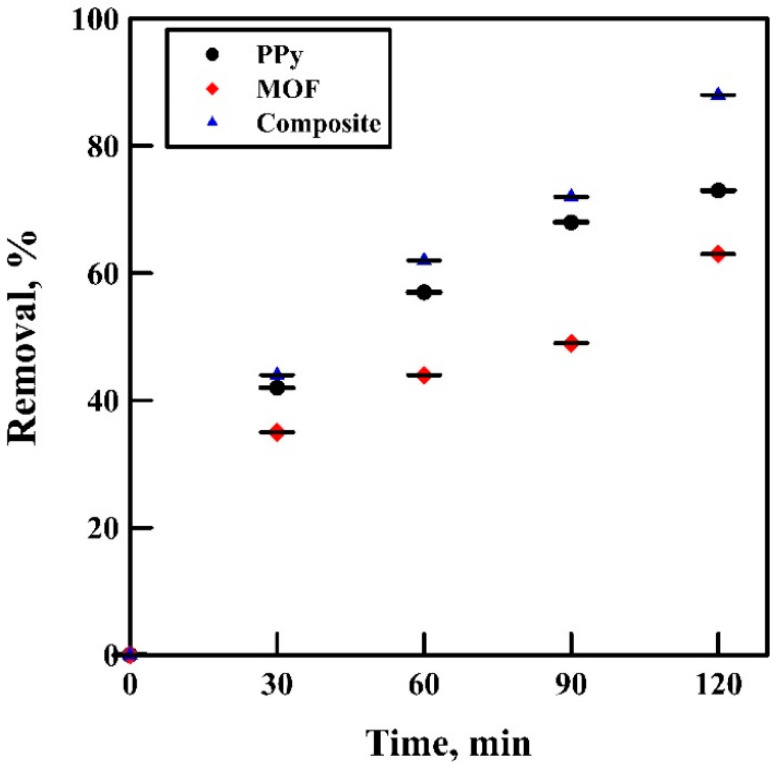
Lead removal efficiencies of PPy, MOF, and composite.

To test the lead removal efficiencies of polypyrrole, MOF, and composite, adsorption experiments at different time intervals have been conducted. The decontamination percentages show that the aluminum fumarate MOF removal efficiency is the least throughout the experiment, and the maximum removal was approximately 60% after two hours. After 30 min, the lead removal by PPy and composite was almost the same, but the removal gap started after an hour. As shown in Figure 12, after two hours, the removal efficacies were 77% and 92% for PPy and composite, respectively. 

### 3.6. Desorption Study

**Figure 13:**
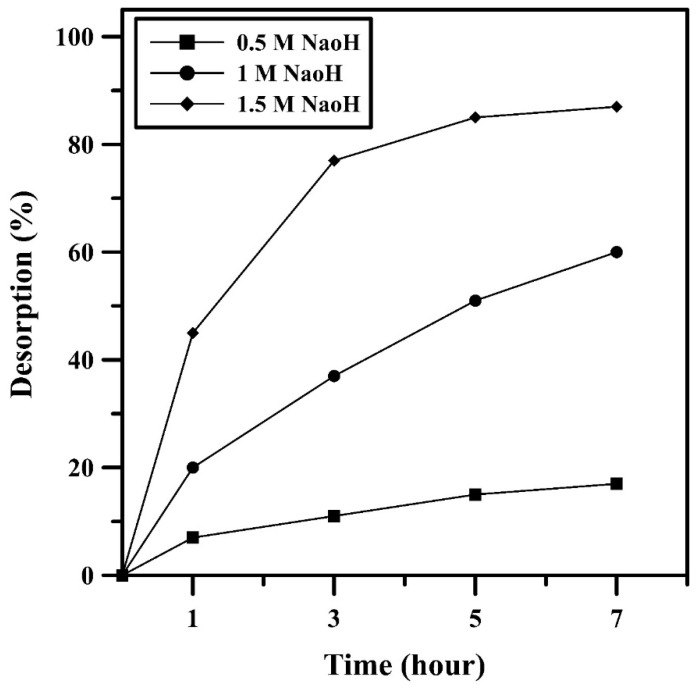
Composite regeneration using NaOH.

The lead desorption percentage is calculated from Equation (5), and the data were plotted against time using different concentrations of sodium hydroxide (0.5, 1, and 1.5 M NaOH). After seven hours, the regeneration percentages of the composite using 0.5, 1, and 1.5 M NaOH were 20%, 61%, and 92%, respectively. Figure 13 suggests that the composite could be regenerated to be recycled by using 1.5 M NaOH for a seven-hour desorption process. 

## 4. Conclusions

Polypyrrole/aluminum fumarate metal–organic framework polymer–MOF hybrid composites were successfully synthesized via in situ polymerization and comprehensively investigated. While the XRD technique shows the presence of the characteristic peaks of both polypyrrole and MOFs at the composite diffraction pattern, BET analysis data proves that the composites have a relatively high surface area, equal to 809 $m^{2}/g$. Furthermore, the composites show lower weight losses than MOF at temperatures up to 470 ℃. The larger surface area of composites has the advantage of better adsorption efficiency, which reaches approximately 92% when removing lead (50 ppm) from water effluent. These results display novel, promising nanocomposites with potential use in lead removal by adsorption process. 

## Figures and Tables

**Table 1:** Langmuir and Freundlich isotherms data.

	Langmuir Isotherm		Freundlich Isotherm	
Temperature K	K	Q_m_	K_f_	*n*
**298**	0.015	600	43	2.63
**308**	0.012	527	38	2.7

**Table 2:** Adsorption capacity comparison between the prepared PPy/MOF and various fabricated AC materials.

Adsorbent Material	Q_e_ (mg/g)	Reference
PPy/MOF composite	600	Present study
Aluminum fumarate MOF	431	[26]
NMAC fabricated using water hyacinth roots	30.20	[32]
AC fabricated using Delonix regia pods	24	[33]
AC fabricated using wheat shells	16.56	[34]
AC fabricated from coconut husk	5.87	[35]
